# Heightened respiratory brain pulsations indicate fluid dynamic dysfunction in early multiple sclerosis

**DOI:** 10.1177/0271678X261453802

**Published:** 2026-05-28

**Authors:** Jere Haverinen, Mervi Ryytty, Harri Rusanen, Johanna Krüger, Mika H Martikainen, Janne Kananen, Lauri Raitamaa, Niko Huotari, Matti Järvelä, Johanna Tuunanen, Heta Helakari, Emma Hiukka, Vesa Korhonen, Vesa Kiviniemi

**Affiliations:** 1 Oulu Functional Neuroimaging, (OFNI), Research Unit of Health Sciences and Technology, Faculty of Medicine, University of Oulu, Oulu, Finland; 2 Diagnostics, Medical Research Center Oulu, Oulu University Hospital and University of Oulu, Oulu, Finland; 3 Research Unit of Clinical Medicine, Neurology, University of Oulu, Oulu, Finland; 4 Neurocenter, Neurology, Oulu University Hospital, Oulu, Finland; 5 Clinical Neurophysiology, Oulu University Hospital, Oulu, Finland; 6 Biocenter Oulu, University of Oulu, Oulu, Finland

**Keywords:** Brain pulsations, CSF dynamics, MREG, blood flow, respiration

## Abstract

Multiple sclerosis (MS) is characterised by perivascular space inflammation and perivenous neuronal demyelination. Cerebrospinal fluid (CSF) is driven along these perivascular structures by vasomotor waves, respiratory and cardiovascular pulsations which also drive cerebral blood flow. As altered blood flow precedes lesion development in vivo, we used fast functional magnetic resonance imaging (fMRI) at 10 Hz and whole-brain statistical maps to investigate whether 25 patients without disease-modifying therapies and with recently diagnosed multiple sclerosis showed alterations in three physiological brain pulsations that drive brain fluid dynamics, compared to 25 age- and sex-matched healthy controls. In this observational study we show that respiratory pulsation’s power (PP_Resp_) is significantly higher in CSF spaces and widely in MS brain compared to the healthy controls. Neither arterial cardiovascular pulsations nor vasomotor waves showed significant differences between the groups. Furthermore, power of respiratory pulsation correlated with greater neurological disability especially in sensorimotor areas and thalamus, and with the higher MS lesion burden. The duration of MS correlated with the increased width range of variability in the autonomous respiratory rate. These findings indicate altered perivenous CSF and intravenous flow dynamics, consistent with the perivenous disposition of MS, and may provide target for new therapies.

## Introduction

Multiple sclerosis (MS) is a potentially disabling chronic neuroinflammatory disease of the central nervous system (CNS) that imposes significant individual burden and ranks seventh to 14th globally in disability-adjusted life-years of all neurological disorders.^1^ Despite decades of research, the specific pathophysiological mechanisms and etiology of MS remain unclear. Brain pathology in MS is diffuse, such that the demyelinating lesions observed in magnetic resonance (MR) imaging represent only a conspicuous component of the disease pathology.^2^ Detectable MS lesions often surround and track venous anatomy in the grey matter, and especially in periventricular white matter, and bear some uncertain relation to structural modification of blood vessels.^3–6^ Changes in cerebral haemodynamics are generally present in MS, and, are known to precede both MS lesion formation^6–9^ and enhancement^10^ that reflects a breach in the integrity of blood–brain barrier (BBB). Furthermore, MS lesions tend to develop in vascular watershed areas that are more susceptible to hypoxia.^7^

Given these associations, MS plaques unsurprisingly bear some resemblance to ischaemic foci, namely with respect to over-expression of aquaporin-4 water channels (AQP4) in the astrocytes delineating the lesion.^11–13^ Astrocyte endfeet with AQP4 channels also delineate perivascular spaces in the brain and are constituting the BBB.^14,15^ Enlarged extralesional and lesional perivascular spaces are a common finding in MS brain being often, but not in all lesion stages, infiltrated with immune cells.^2,16–20^

Blood flow dynamics, the flow of brain interstitial fluid (ISF) and cerebrospinal fluid (CSF) are driven by neuronal activity^21^ and a triad of physiological pulsations, namely cardiovascular, respiratory and slow vasomotor pulsations,^22,23^ which were first described by Berger.^24^ At the microscopical level in brain, cortical venous structures pulsate in concert with breathing and cardiovascular impulses, which also drive periarterial and arterial pulsation, which is modulated by relatively slow vasomotor waves.^25–28^ According to the current knowledge, there are distinct perturbations of the triad of physiological brain pulsations in diverse neurological conditions, such as epilepsy (respiratory pulsation), Alzheimer’s disease (cardiovascular pulsation), narcolepsy (slow vasomotor pulsation and cardiovascular pulsation) and primary central nervous system lymphoma, but physiological changes in the whole triad of pulsations occur during sleep and in relation to vigilance.^29–33^ These local brain pulsation perturbations likely have pathophysiological relevance to brain fluid dynamics. The prior findings indicate that brain pulsation perturbation could lie within veins or perivascular spaces filled with fluid and immune cells in MS. Assessing that requires methods that can capture fluid movement at cardiorespiratory rates. An ‘ultrafast’ fMRI sequence known as magnetic resonance encephalography (MREG) can distinguish simultaneously acquired ~1 Hz arterial flow impulses from venous blood oxygenation level-dependent (BOLD) pulsations, that are driven by ~0.3 Hz respiration and very low frequency (<0.1 Hz VLF) vasomotor waves, without aliasing due to its high frequency (10 Hz) sampling for the entire brain.^27,34^

The MREG BOLD signal is generally attributed to susceptibility changes in water proton spins inside and around the brain blood vessels due to changes in the local concentration of paramagnetic deoxyhaemoglobin concentration that arise secondary to blood flow changes arising due to neurovascular coupling^28,35–38^ and respiratory changes.^33,39,40^ Previous literature on slower functional magnetic resonance imaging (fMRI) scannings indicates that respiratory breath volume and end-tidal CO_2_ correlate strongly with the BOLD signal.^39,40^

The cardiovascular pulsations (~1 Hz) in MR signal are induced by water molecule accelerations that induce spin phase changes and steady-state free precession (SSFP) spin-coherence perturbations especially in and around arteries, the sagittal sinus and CSF.^26,28,41–43^ Cardiovascular (arterial) pulsations are modulated by both vasomotor waves and respiration.^26,28,44^ Respiratory rate (~0.3 Hz) MR signal pulsations originate from CSF flow and intravenous blood outflow of deoxyhaemoglobin-driven BOLD signal changes induced by respiratory pressure changes inside thorax and spinal canal.^26,33,39,40,43^ CSF does not produce a classical BOLD signal as it contains no (de)oxyhaemoglobin, but in slice imaging data CSF is known to have both T1 and T2 phase effects and time-of-flight effects due to flow.^45^ The relative contributions of those effects are more complex in the 3D spiral acquisitions, such as MREG.^34,43^ However, pulsatile water flow has been shown to induce verifiable MREG signal oscillations arising from both the frequency and speed in a water flow phantom experiment.^46,47^

The perivenous proclivity of MS plaques suggests an altered flow either in veins and/or in perivenous CSF spaces. Thus, we aimed to investigate the relationship of plausibly altered flow and perivenous disposition of MS. To test this hypothesis of altered flow, we compared the power of physiological brain pulsations, which drive both blood and perivascular CSF, in early non-medicated MS versus age- and sex-matched controls using non-invasive whole brain MREG data. In keeping with the perivenous disposition of MS, we detected significant individual and group-level differences in the power of physiological brain pulsations reflecting altered venous blood and CSF flow.

## Material and methods

### Subjects and ethics

We adhered the relevant STROBE checklist for observational studies to ensure transparent and accurate reporting. The study cohort initially comprised 32 volunteer patients, who were recruited and examined between 2018 and 2021 by experienced neurologists at the Department of Neurology of Oulu University Hospital (Oulu, Finland), with a recent diagnosis of either clinically isolated syndrome or MS. Among this group, 25 patients met our final inclusion criteria for further analysis: diagnosis of multiple sclerosis according to 2017 McDonald criteria, acceptable MREG data quality (no missing brain voxels or exceptional drifts in filtered signal to visual inspection) and no initiation of disease-modifying therapies for MS.

Use of medications such as corticosteroids, or the presence of additional CNS or vascular diseases could potentially affect BBB or brain pulsation physiology. Comorbidities are known to be common in MS.^48^ Therefore, we assessed all medication regimens and comorbidities of MS patients. (see Table 1 for demographics)

**Table 1.** Demographics.

Characteristic	MS (*n* = 25), median (IQR; min–max^a^; sum of ranks)	Controls (*n* = 25), median (IQR; min–max^a^; sum of ranks)	*p* value (*q*-value)^b^
Age (years)	36.1 ± 8.02 (21–51)^a^, *t* = 0.285	35.5 ± 7.88 (21–48)^a^, *t* = 0.285	0.78 (0.67)
Males (*n*)	9 (36%)	9 (36%)	
Females (*n*)	16 (64%)	16 (64%)	
Smoking (*n*)	7 (28%)	2 (8%)	
Mean absolute head displacement (mm)	0.15 (0.13–0.29; 0.099–0.48; 615)	0.17 (0.14–0.24; 0.060–0.42; 660)	0.67 (0.64)
Mean relative head displacement (mm)	0.034 (0.027–0.043; 0.020–0.087; 725)	0.029 (0.025–0.035; 0.020–0.053; 550)	0.092 (0.18)
Respiratory rate (Hz)	0.25 (0.18–0.31; 0.11–0.43; 607)	0.28 (0.22–0.31; 0.090–0.37; 668)	0.56 (0.62)
Heart rate (Hz)	1.1 (1.0–1.3; 0.90–2.0; 750.5)	1.0 (0.97–1.1; 0.85–1.5; 524.5)	0.028 (0.079)
MS characteristics			
Days from MS diagnosis to MREG^c^	26^d^ (12–43; −106 to 727)		
Months from the first symptom to MREG^e^	9.5 (5–60; 1–201)		
Days from EDSS evaluation to MREG^f^	15^d^ (5–28; −436 to 61)		
EDSS	2.0 (0.5–2.5; 0–6.5)		
EDSS <2.0 (*n*)	12 (48%)		
EDSS ⩾2.0 (*n*)	13 (52%)		
RRMS (*n*)	24 (96%)		
PPMS (*n*)	1 (4%)		
Patients with T1 gadolinium-enhancing lesion ± 4 weeks from MREG (*n*)^g^	6 (24%; 13 (52%) had a recent gadolinium-enhanced scanning)		
Relapse during or 3 months prior to MREG (*n*)	11 (44%)		
Corticosteroid treatment 3 months prior to MREG (*n*)	5 (20%)		
No medications (*n*)	8 (32%)		
No other medication than vitamin supplements (*n*)	10 (40%)		
Disease-modifying therapies (*n*)	0 (0%)		
CNS targeting medication (*n*)^h^	11 (44%)		
Vitamin D (*n*)	9 (36%)		
MS T2FLAIR lesions			
1–15 lesions (*n*)	14 (56%)		
16–29 lesions (*n*)	6 (24%)		
⩾30 lesions (*n*)	5 (20%)		
Patients with brain stem lesion (*n*)	14 (56%)		
Comorbidities (*n*)			
No comorbidities, no medication, no corticosteroid	7 (28%)		
⩾2 comorbidities	9 (36%)		
Epilepsy	2 (8%)		
Migraine with aura	2 (8%)		
Depressive episode or disorder	7 (28%)		
Panic or anxiety disorder	3 (12%)		
Sleep apnea	1 (4%)		
Cold urticaria or cold allergy	1 (4%)		
Hypertension (essential or secondary)	1 (4%)		
Graves disease	1 (4%)		
Hypothyroidism	3 (12%)		
Type 2 diabetes mellitus	1 (4%)		
Asthma	4 (16%)		
Crohn disease	1 (4%)		
Gastro-esophageal reflux disease	2 (8%)		
Other diseases of liver	1 (4%)		
Hallux rigidus	1 (4%)		
Gout	1 (4%)		

MS: multiple sclerosis; RRMS: relapsing-remitting multiple sclerosis; PPMS: primary progressive multiple sclerosis; MREG: magnetic resonance encephalography; FLAIR: fluid-attenuated inversion recovery; EDSS: Expanded Disability Status Scale; CNS: central nervous system; FDR: false discovery rate.

Subject demographics at the time of MREG scanning.

a Values represent median, interquartile range, range from minimum to maximum and sum of ranks in Mann–Whitney test, or number of subjects and corresponding frequency. Mean ± corresponding standard deviation are reported instead of median, and *t* = *t*-ratio instead of sum of ranks, when the distribution’s skewness was in the range −0,5 < *X* < 0,5.

b *p* values represent FDR corrected exact two-tailed Mann–Whitney tests, except *p* value of the age distribution difference, which represents an unpaired *t*-test. Utilised FDR correction is two-stage linear step-up procedure of Benjamini, Krieger and Yekutieli with *Q* = 5%.

c Negative value indicates that MREG imaging was conducted before definitive MS diagnosis. Positive value indicates that MS diagnosis was made before MREG scanning.

d Absolute values of date difference were used to calculate median and interquartile range.

e Time since the first MS symptom was available from 24 patients with an accuracy of month and year. Though the duration from the first symptom was relatively long in several cases, it can take years before MS diagnosis can be set.

f The EDSS evaluation date was indeterminate in one case. Negative value indicates that closest EDSS evaluation was after MREG scanning. Positive value indicates EDSS evaluation prior to MREG scanning.

g Contrast enhancement usually persists around 1 month.

h Antidepressant, levetiracetam, carbamazepine, pregabalin/gabapentin, tizanidine, amantadin, quetiapine or oxazepam.

We selected an age- and sex-matched healthy control dataset for every MS patient. Recruitment criteria for the volunteer controls included self-reported absence of medication other than non-opioid analgesics or contraceptives and acceptable MREG data quality. To control potential confounders, all subjects reported their current smoking status (yes/no) and consumption of coffee, other stimulants, alcohol, cannabis and other intoxicants within 12 h prior to MREG. No patients reported consumption of cannabis, stimulant drugs or narcotics prior to imaging. Notably, most subjects had consumed coffee or other sources of caffeine, with similar consumption between the groups.

Screening included evaluation of each subject’s MRI safety. An experienced neuroradiologist (VKi) reviewed each subject’s anatomical images for unexpected brain pathology. Written informed consent was obtained from all participants in accordance with the Declaration of Helsinki guidelines. The study was approved by the Regional Ethics Committee of the Northern Ostrobothnia Hospital District (53/2012, 274/2020).

### MRI data acquisition and preprocessing of MREG data

All subjects were scanned at the Oulu University Hospital between 2018 and 2021 using a Siemens 3T magnetic resonance imaging (MRI) scanner (Siemens Healthineers AG, Germany). Lesion load was determined by manual quantification of MS plaques from anatomical T2-weighted-Fluid-Attenuated Inversion Recovery (T2FLAIR, a.k.a. Dark Fluid, DF, in Siemens scanners) images, performed alongside an experienced neuroradiologist (VKi). Also, all gadolinium (Dotarem® 279,3 mg/ml i.v.) contrast-enhanced T1-weighted images acquired ± 4 weeks from MREG were evaluated to detect disease activity. MREG was either conducted simultaneously or close to a diagnostic or follow-up anatomical imaging, and MRI contrast was used only if there was a clinical indication (see Table 1).

MREG, a 3D single shot stack of spirals sequence, under-samples k-space to achieve a 10 Hz sampling rate, capturing the entire brain volume with single brief excitation. Scanning parameters for MREG sequence were repetition time (TR) 100 ms, echo time (TE) 36 ms, flip angle = 25°, 3D matrix = 64^3^, FOV = 192 mm with voxel size of 3 × 3 × 3 mm^3^. Furthermore, the magnetisation spoiling gradient between scans was set to 0.1 to avoid signal masking by stimulated echo drifts, while retaining sensitivity to physiological pulsations. For anatomical T1-weighted 3D MPRAGE, the parameters were TR = 1900 ms, TE = 2.49 ms, TI = 900 ms, flip angle = 9°, FOV = 240 mm, 0.9 mm cubic voxel. MREG data reconstruction, artefact control and preprocessing are described precisely in the Supporting Information. Using anatomical T1-weighted 3D images, MREG data were registered into Montreal Neurological Institute (MNI152) standard space. Five-minute resting state fMRI scanning protocol yielded 2961 brain volumes. Subjects were instructed to lie still with their eyes open and with gaze fixation on a cross displayed on a screen. Soft pads and ear plugs were fitted over the subjects’ ears to dampen auditory stimuli and minimise head motion.

For image reconstruction and preprocessing, we utilised FSL (Functional Magnetic Resonance Imaging of the Brain’s software library) and MATLAB (The MathWorks, Inc., MA, USA) software, with additional processing in Analysis of Functional NeuroImages (AFNI).

### Power spectrum analysis of physiological MREG signals

**Figure 1.**
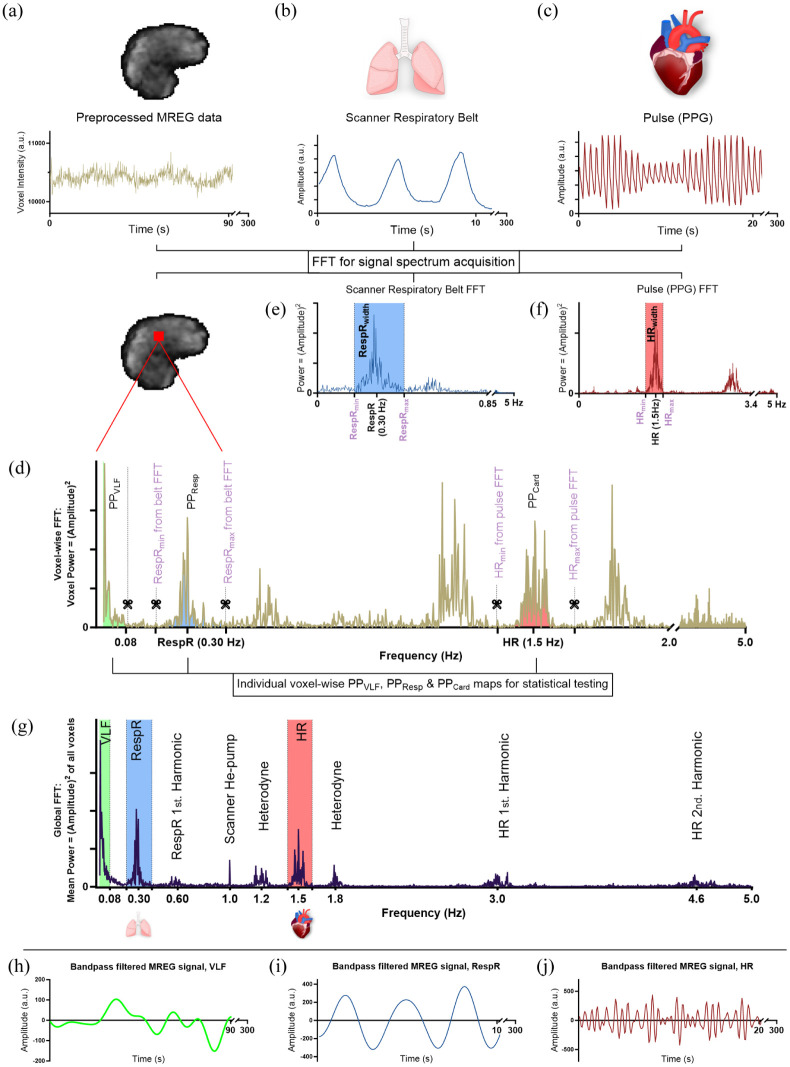
Power spectrum analysis of physiological MREG signals and demonstration of MREG signal frequency components: (a–f) data analysis pipeline after MREG image reconstruction, brain extraction and image preprocessing, (d) very low frequency, respiratory and cardiac frequency bands were extracted from MREG voxel-wise FFT data for statistical analysis (representative subject data are shown). Respiratory and cardiac frequency bands (e, f) were verified from external cardiorespiratory monitoring data (b, c). The signal power is proportional to the square of the amplitude for a given frequency, (g) an example global MREG FFT spectrum showing different spectral components, and illustrating that spectral component cutoff values are equal to the cardiorespiratory monitoring data (e, f). We carefully avoided in our analysis heterodynes, which equal HR ± RespR, and harmonic frequencies, which are also evident in respiration belt and PPG FFT spectra (e–g), and (h–j) examples of band-passed MREG signals to differentiate very low (h), respiratory (i) and cardiac (j) signal frequency components. We determined the frequency ranges for band passing from the cardiorespiratory monitoring data FFT periodograms. MREG: magnetic resonance encephalography; FFT: fast Fourier transform; PPG: photoplethysmograph; RespR: principal respiratory rate; HR: principal heart rate; PP: power of pulsation; VLF: very low frequency band; Resp: respiratory frequency band; Card: cardiac frequency band (i.e. arterial pulsation impulses).

We depict our approach for analysis of brain physiological pulsation power from preprocessed MREG data in Figure 1. Our power spectrum analysis is similar to the well-known amplitude of low frequency fluctuations (ALFF) method,^49^ with calculation of power rather than amplitude. Furthermore, ‘ultrafast’ MREG enables detection of physiological signal fluctuations up to 5 Hz without aliasing. Here, the frequency band for PP_VLF_ corresponds to the classical ALFF. In the PP (power of pulsation) maps, a voxel value represents the total voxel-wise power of a chosen frequency band (VLF, respiratory or cardiac). The power is proportional to the square of the amplitude of a given frequency, and the BOLD signal fluctuation in a given voxel on a chosen frequency band reflects the local features of brain oscillatory activities.^49–52^

To verify and monitor individual respiratory (RespR) and heart rate (HR), we recorded scanner respiration belt and finger SpO_2_ photoplethysmograph (PPG) data simultaneously with MREG. Indeed, the MREG pulsation data including RespR and HR precisely matched to the simultaneously monitored cardiorespiratory data (see S3 Figure in Supplementary File).^27,32^ For every subject, we transformed the time domain signal of each 3^3^ mm^3^ voxel from the whole brain MREG data into a voxel-wise power spectrum using AFNI *3dPeriodogram*, which employs the Fast Fourier Transform (FFT) algorithm. We then obtained individual whole brain, that is, global, power spectra by averaging all brain voxels with *fslmeants* and then further averaged all individual global spectra to get the mean global MREG power spectra for the MS and control groups.

From whole brain voxel-wise MREG power spectra, the power of pulsation (PP) frequency bands of vasomotor very low frequency (PP_VLF_, 0.008–0.08 Hz for all subjects), individual respiratory pulsation frequency (PP_Resp_) and cardiovascular, that is, cardiac arterial pulsation frequency (PP_Card_) were separated and individually summed in each voxel to obtain corresponding PP maps (three per subject). To accurately separate PP_Resp_ and PP_Card_, before summation we visually verified the RespR and HR spectral component frequency bands from minimum to maximum frequency power above baseline noise from each subject’s cardiorespiratory monitoring data FFT spectra, as first calculated in MATLAB (Figure 1). RespR_width_ and HR_width_, indicating, respectively, the variability ranges of respiratory and heart rates, were calculated as the difference between the measured minimum and maximum frequencies. For data visualisation, whole-brain (WB), that is, global and structural (grey matter = GM, white matter = WM, CSF) mean PP values, equal to image mean pulsation power intensity, were calculated from individual MS PP_(VLF/Resp/Card)_ maps in reference to Harvard-Oxford Subcortical Structural Atlas.

### Statistical methods

Two-way comparisons with *p* ⩽ 0.05 was deemed statistically significant. Voxel-wise between-group differences in processed MREG pulsation data were examined using widely used nonparametric FSL’s *randomise* tool (general linear model (GLM)) with 10,000 Monte Carlo random permutations implementing family-wise error-corrected threshold-free cluster enhancement corrections, adjusted for mean relative head displacement.^53–55^ This yielded statistical images with corrected *p* values as shown in the Results section.

Medication and comorbidities in our entire sample of MS patients (detailed in Table 1) were included in our statistical models. To assess if certain covariates are related to the power of brain respiratory pulsation (PP_Resp_) in the MS group, we undertook voxel-wise statistical analyses between patients with and without additional diagnosed neurological or psychiatric disorders, use of centrally-acting medication, relapse (including corticosteroid therapy) within 3 months prior to MREG scanning, EDSS 2.0 or greater, more than 15 T2FLAIR lesions, or presence of at least one brainstem lesion. These cutoffs were chosen because brainstem lesions could affect the brain’s respiratory centers, thus potentially causing dominant pulsation abnormalities. Moreover, EDSS 2.0 is generally considered a cutoff between minimal signs (no disability) and minimal disability, and corticosteroid therapy could affect BBB for weeks. These contrasts with nearly equal group sizes, were performed individually in FSL’s *randomise* tool, with the options described earlier, and adjusted for the mean relative head displacement. Thus, we examined whether a few subjects with the covariate could account for the pulsation power differences between MS and controls specifically in this sample. The independent effect of covariaties (RespR_width_ and smoking status) and statistical images showing brain regions with a linear relationship between PP and EDSS were also computed with *randomise*. Thus, we were able to assess whether these covariates explained observed differences in PP between the groups and if neurological impairment correlated with PP values in some brain regions within the MS group.

MREG power of pulsation results between the MS and control groups were adjusted for brain structural and volumetric differences by using voxel-based morphometry (VBM) technique. Structural data was analysed with FSL–VBM,^56,57^ an optimised VBM protocol.^58,59^ First, structural T1 images were brain-extracted and GM-, WM- and CSF-segmented before being registered to the MNI152 standard space using non-linear registration.^60^ The resulting images were averaged and flipped along the *x*-axis to create a left-right symmetric, study-specific GM, WM and CSF templates. Second, all native GM, WM and CSF images were non-linearly registered to their respective study-specific template and ‘modulated’ to correct for local expansion (or contraction) due to the non-linear component of the spatial transformation. The modulated GM, WM and CSF images were then smoothed with an isotropic Gaussian kernel with a sigma of 3 mm. Finally, voxelwise GLM (*randomise*) was applied using permutation-based non-parametric testing, correcting for multiple comparisons across space.

Furthermore, we combined fMRI and structural analysis to calculate voxel-wise which brain areas have a linear relationship between power of brain pulsations and relative CSF volume. Relative CSF volume reflecting the extent of central and cortical atrophy was calculated by dividing subject’s CSF volume with the sum of CSF, GM and WM volumes. Dividing by whole-brain volume normalises for inter-subject differences in normal CSF volume. The ratio increases in atrophy, as a loss of brain tissue leads to an expansion of CSF spaces. Segmented cortical CSF includes sulcal CSF only and does not encompass the full subarachnoid space; however, enlarged sulci still reflect cortical atrophy and it aligns with the masking of MREG data.

To detect individual distribution of significant pulsation differences in MS, we calculated the *Z*-score of each voxel for six patients with *fslmaths.* Thus, we created statistical images with *Z*-values using the corresponding voxel’s mean and standard deviation (SD) value from the averaged PP map of 25 healthy controls. In addition, we calculated the intrasubject *Z*-score of each voxel using the subject’s individual whole brain mean PP and SD values. The six selected patients represent a range from low to huge image mean intensity, that is, the mean PP values, in our study sample. For display purposes, all computed statistical images in MNI152 standard space were interpolated to higher resolution in FSLeyes.

Further statistical analyses were conducted in Prism 9 (GraphPad, San Diego, CA, USA) to control for potential covariates and support voxel-wise analyses. Descriptive statistics and histograms were calculated first. After testing for normality, the group’s cardiorespiratory frequencies, age distribution, head displacement values, WB and structural mean PP values were compared with two-tailed *t*-test or exact two-tailed Mann–Whitney test. Exact Mann–Whitney test was chosen if either one of variables did not pass both D’Agostino and Pearson test and Shapiro–Wilk test for normality, or if the histogram appeared non-Gaussian to visual inspection. Under those circumstances, medians and interquartile ranges were reported instead of means and standard deviations, which were reported for normally distributed data (see Table 1). Frequencies were reported for categorical data. The measured data rarely followed the normal distribution. Thus, relations among MS clinical characteristics, mean PP and head displacement values were investigated with Spearman correlation coefficients, as outlined in the Results section.

*Alpha* = 0.05 was deemed statistically significant. Tests calculated in Prism were grouped into families and all *p* values were corrected for the false discovery rate (FDR). Between-group comparisons (MS vs controls) formed the primary family and 25 *p* values, including *p* values from correlations, were corrected with Two-stage linear step-up procedure of Benjamini, Krieger and Yekuteli method with *Q* 5%. Associations between structural mean PP-values with clinical variables within the MS group were treated as a separate family of secondary hypotheses. The same method was used to correct 30 *p* values in this family but 10% *Q* was accepted in order to balance between type I and type II error in a relatively small sample of 25.

## Results

### Demographics

Clinical characteristics of the patients were obtained from their medical records, and all diagnoses were re-evaluated by experienced neurologists (JKr, MR and HR; for more detailed information on subjects; see Table 1). We conducted MREG scanning of the patients (mean age 36.1 ± 8.02 years, females 16/25, relapsing remitting MS (RRMS) 24/25, primary progressive MS (PPMS) 1/25 (confirmed later)) with a median time of 9.5 months after the first symptom of MS, and in 75% of the cases within 43 days from the MS diagnosis (median 26 days (min −106 days, max 727 days)). Each MS patient underwent an Expanded Disability Status Scale (EDSS) evaluation by a neurologist, with evaluation of 75% of cases within 28 days of the MREG scanning, yielding a median EDSS score of 2.0.

There were no MS cases receiving disease-modifying therapies. Only five patients had received methylprednisolone therapy within 3 months prior to MREG. Eleven patients had one or more CNS-targeting medication in daily or in occasional use. Four MS patients had an additional neurological condition (epilepsy, migraine with aura). Eight patients had a psychiatric disorder (depressive episode or disorder, panic or anxiety disorder) and one had sleep apnea (see Table 1). While hypertension could affect physiological brain pulsations, especially the cardiac pulsation, only one MS patient was diagnosed with hypertension.

Compared to the healthy controls (*n* = 25, mean age 35.5 ± 7.88 years, females 16/25), the age distribution (*p* = 0.78, *q* = 0.67, *t* = 0.285), mean absolute and mean relative head displacement values (*p* = 0.67, *q* = 0.64 and *p* = 0.092, *q* = 0.18, respectively) and respiratory and heart rate (*p* = 0.56, *q* = 0.62 and *p* = 0.028, *q* = 0.079, respectively) in the MS group did not statistically differ (see Table 1 for group details and statistics).

### Brain pulsation power analytics – Respiratory pulsations were heightened in MS patients

**Figure 2.**
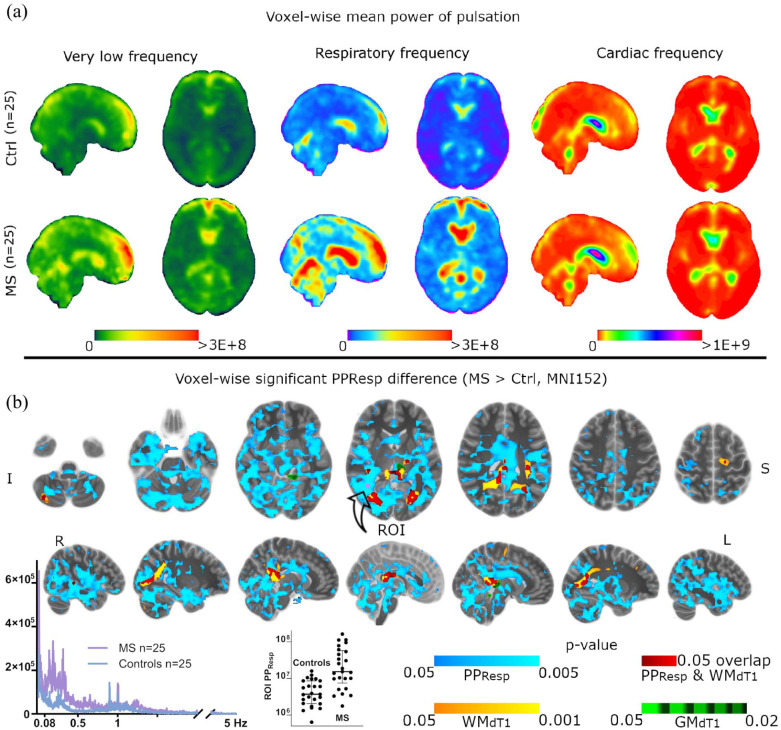
Physiological brain pulsation differences between MS and healthy control groups: (a) axial and sagittal slices of voxel-wise control and MS group mean PP_VLF/Resp/Card_ maps. Generally, MREG signal power is highest in the ventricles and other CSF-spaces. Note that ACA and MCA perimeters stand out as yellow in the cardiac frequency axial slice and (b) image slices show voxels with significantly greater power of respiratory frequency pulsation, adjusted for mean relative head displacement in the MS compared to healthy controls. Colours represent FWE-corrected *p* values. PP_Resp_ was significantly higher in MS compared to controls in various brain regions including the lateral ventricles, subcortical structures, grey matter and brainstem respiratory centers. Pneumotaxic center and medullary respiratory centers are seemingly connected by a structural line of increased PP_Resp_. Yellow shows areas with the greater white matter atrophy and green shows areas with the greater grey matter atrophy, or T1 signal hypointensity, in our MS sample compared to the healthy controls. Red shows the volumetric overlap of heightened PP_Resp_ difference and the white matter atrophy (T1 signal hypointensity) difference between MS and control groups, which is 2.4%. Global MREG FFT periodograms averaged over whole groups showing greater power at physiological respiratory frequencies (~0.3 Hz) and wider respiratory spectral component width, that is, respiratory rate variability range (RespR_width_) as a plot. The physiological cardiac spectral component lies around 0.8–1.3 Hz. PP_Resp_ values between the groups from a ROI, and group median with interquartile range. Note logarithmic *y*-axis. Background: MNI152 standard template. MS: multiple sclerosis; Ctrl: control; PP: power of pulsation; VLF: very low frequency band (0.008–0.08 Hz); Resp: respiratory frequency band; Card: cardiac frequency band; WM_dT1_: white matter atrophy or T1 signal hypointensity in MS > controls; GM_dT1_: grey matter atrophy or T1 signal hypointensity in MS > controls; CSF: cerebrospinal fluid; MREG: magnetic resonance encephalography; ACA: anterior cerebral artery; MCA: middle cerebral artery; I: inferior; S: superior; R: right; L: left; FFT: fast Fourier transform; ROI: region of interest.

Neither cardiac (arterial frequency) pulsations nor vasomotor VLF wave power showed significant differences between MS patients and healthy controls on a group level to MREG power spectrum analysis in either direction, cf. Figure 2(a). The power of individual respiratory brain pulsations in the MREG data (PP_Resp)_ was significantly higher in the MS group compared to healthy controls (family-wise error-corrected *p* ⩽ 0.05 and *p* ⩽ 0.01; adjusted for mean relative head displacement), Figure 2(a) and (b) and S1 Figure in Supplementary File shows more slices. Heightened respiratory pulsations were observed mainly in subcortical WM, grey matter, periventricular regions, midbrain, pons, medulla, cerebellum, in CSF of the lateral and third ventricles, and from the level of medulla to the most superior parts of the cerebrum. Interestingly, there were very significant group differences in brain respiratory centers: the pneumotaxic center or pontine respiratory group, Dorsal Respiratory Group (DRG) and Ventral Respiratory Group 9 VRG) and in the area lying between. No statistically significant PP_Resp_ differences were seen in the controls > MS direction. Groups’ mean global MREG FFT spectra (Figure 2(b)) show the increased PP_Resp_ and RespR_width_ in the MS group as a plot. The spectral component of respiration is wider and of greater power in the MS compared to the healthy controls.

The structural analysis showed significant WM and GM changes, but no CSF volume changes between MS and control groups. There was a minimal region of GM loss in thalmic region in the group-level, overlapping with the areas of WM atrophy or WM intensity changes. MS group had significant WM atrophy, or T1 signal decrease especially in bilateral posterior periventricular regions and corpus callosum, and more contralateral to the hemisphere which had more PP_Resp_ differences, compared to the control group (Figure 2(b) and S1 Figure). There was only a 2,4% overlap between areas of statistically significant WM atrophy or T1 signal decrease, and areas with heightened PP_Resp_.

**Figure 3.**
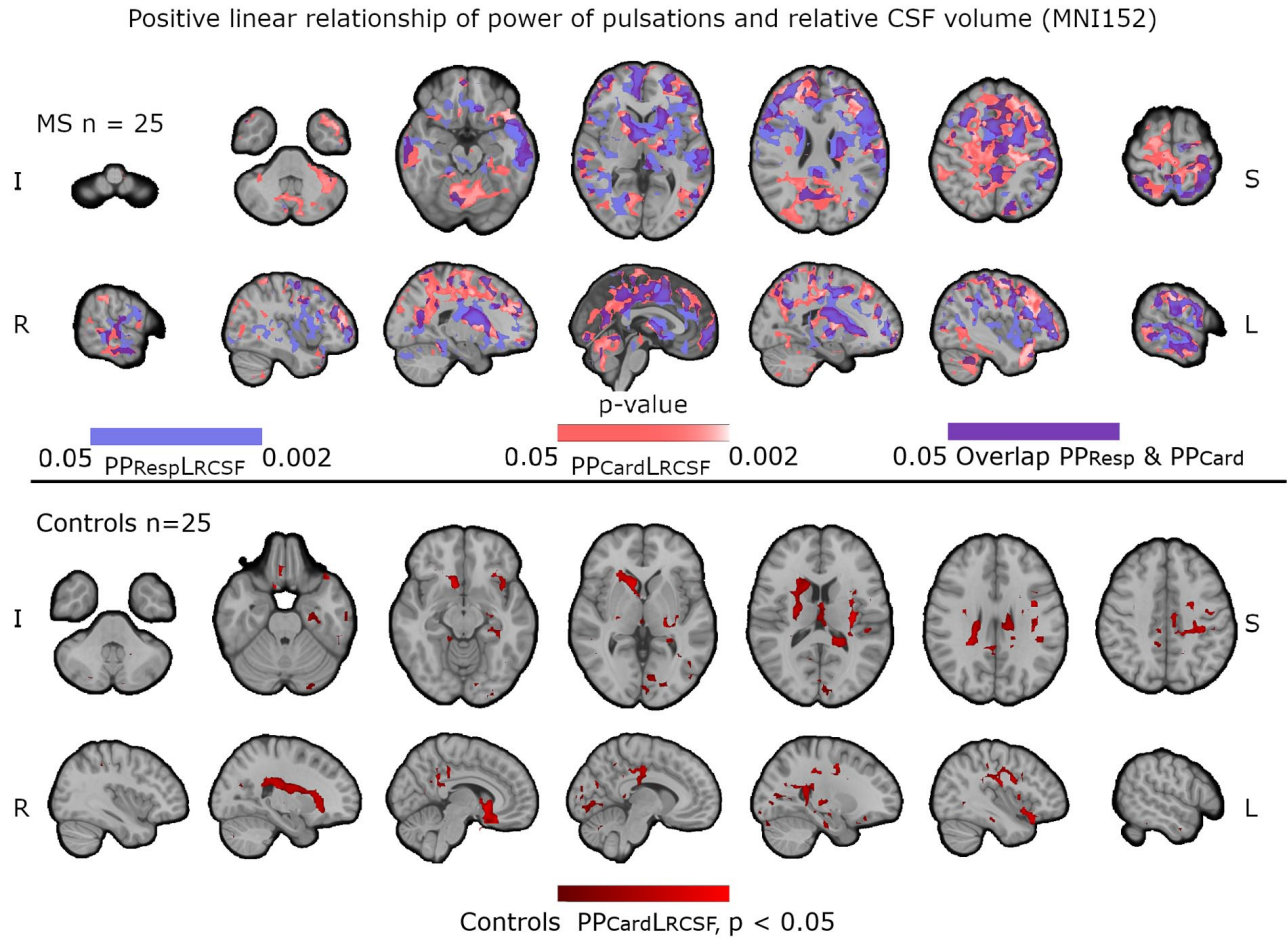
Brain areas with significant positive linear relationship between increased power of respiratory pulsation, cardiovascular (i.e. arterial frequency) pulsation and relative CSF volume increase in MS. The relative CSF volume reflecting the extent of central and cortical atrophy was calculated by dividing subject’s CSF volume with the sum of CSF, GM and WM volumes. Dividing by whole-brain volume normalises for inter-subject differences in normal CSF volume. The ratio increases in atrophy, as a loss of brain tissue leads to an expansion of CSF spaces. Segmented cortical CSF includes sulcal CSF only and does not encompass the full subarachnoid space; however, enlarged sulci still reflect cortical atrophy and it aligns with the masking of MREG data. Increased CSF volume in the ventricles and sulci compared to brain tissue volume, has a positive linear relationship with heightened respiratory and cardiovascular arterial frequency pulsations in MS, and often in the same brain regions. The relationship is evident in brain tissue and periventricular regions but not in the ventricles themselves. Healthy controls displayed the similar relationship in Card pulsation to much lesser extent. (see also S2 Figure). Background: MNI152 standard template. MS: multiple sclerosis; PP: power of pulsation; Resp: respiratory frequency band; Card: cardiovascular (i.e. arterial) frequency band; L_RCSF_: positive linear relationship with relative CSF volume; WM_dT1_: white matter atrophy or T1 signal hypointensity in MS > controls; CSF: cerebrospinal fluid; I: inferior; S: superior; R: right; L: left.

However, there is a positive linear relationship between increased relative CSF volume, thus atrophy, and with the heightened PP_Resp_ and PP_Card_, see Figure 3. Both relationships are widely evident in the MS brain around the group-level atrophic regions. There was 29% overlap in areas with heightened PP_Resp_ in MS versus controls and areas with positive linear relationship of PP_Resp_ and increased relative CSF volume in MS (see S2 Figure in Supplementary File, which also shows more slices). The power of VLF pulsations or PP_Resp_ in controls showed no such positive or negative linear relationships but PP_Card_ increses also in healthy control brain when relative CSF volume is larger. (see Figure 3).

**Figure 4.**
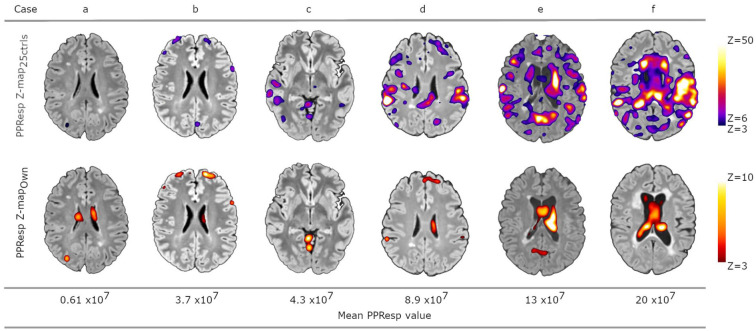
Individual distribution of increased respiratory pulsation power in MS. Intrasubject and control data-based *Z*-maps of six MS patients (age 29–45 years) and the corresponding T2FLAIR axial slice as a function of increasing global mean PP_Resp_ value. Colours represent *Z*-values. Upper row: *Z*-map calculated with control data mean and SD (*n* = 25). Bottom row: *Z*-map calculated with the subject’s own global mean and SD. Individually, most significant areas with increased PP_Resp_ are in CSF filled spaces such as the ventricles and sulci, and (concerning PP_Resp_ signal origin) in perivascular spaces. In most of the visible MS-lesions, PP_Resp_ is not relatively high. Notably, two patients (e, f) having enormous PP_Resp_ (*Z* > 50) had enlarged ventricles. Background: individual fluid-attenuated inversion recovery image. MS: multiple sclerosis; PP: power of pulsation; Resp: respiratory frequency band; SD: standard deviation.

Upon investigation of PP_Resp_ *Z*-maps and anatomical images overlaid upon MNI coordinate space, the highest PP_Resp_ were detected in CSF spaces including the ventricles, sulci or cortical liquor spaces, and (concerning PP_Resp_ signal origin) also in brain tissue perivascular/perivenous spaces, see Figure 4. On visual inspection of *Z*-maps, MS lesions do not coincide with brain areas with relatively high PP_Resp_.

### EDSS has neuroanatomically relevant positive linear relationship with respiratory pulsation power

**Figure 5.**
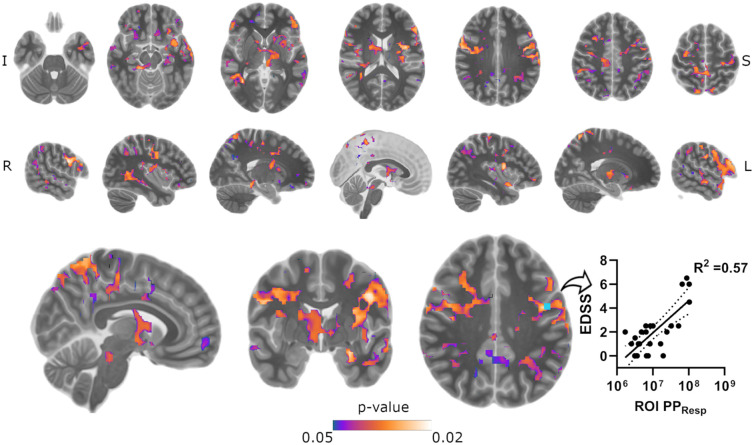
Brain areas having positive correlation of EDSS and power of respiratory pulsation. EDSS score has a significant linear relationship with PP_Resp_ in various brain regions, especially bilaterally in the precentral and postcentral gyrus, adjacent deep structures, thalamus and in the brainstem between pneumotaxic and apneustic center. Colours represent FWE-corrected *p* values. Linear regression and the best fitting curve (semi-logarithmic line, least squares method with 95% CI) of EDSS and PP_Resp_ from a ROI, which is the Talairach coordinate −45 −3 36 in the left primary motor cortex/precentral gyrus. Background: MNI152 standard template. PP: power of pulsation; Resp: respiratory frequency band; EDSS: Expanded Disability Status Scale; I: inferior; S: superior; R: right; L: left; ROI: region of interest.

Figure 5 shows brain areas where EDSS score, which weights motor and sensory function, had a positive linear relationship with PP_Resp_. Thus, when EDSS score was higher, the coloured brain areas had greater MREG signal power at the respiratory rate (family-wise error-corrected *p* ⩽ 0.05). That relationship was mostly evident in grey and white matter in the sensorimotor network area, thalamus, capsula interna and, interestingly, a zone between the pontine respiratory centers. Ventricular CSF showed only a few voxels having a significant correlation. There were no brain areas where PP_Resp_ had a significant negative linear relationship with EDSS.

### Power of respiratory pulsation correlates with clinico-radiological disease appearance in MS

For the MS group, the mean respiratory PP value (= respiratory PP map’s mean intensity) of whole brain (WB; *p* = 0.0001, *q* = 0.001, sum of ranks 828 and 447), white matter (WM; *p* = 0.0003, *q* = 0.002, sum of ranks 819 and 456), grey matter (GM; *p* = 0.0001, *q* = 0.001, sum of ranks 829 and 446) and ventricles, that is, CSF (*p* = 0.0006, *q* = 0.003, sum of ranks 809 and 466 in lateral ventricles, *p* = 0.0037, *q* = 0.015, sum of ranks 785 and 490 in fourth ventricle) was significantly higher compared to controls. There were no significant differences in corresponding mean PP_VLF_ or mean PP_Card_ values between the two groups. However, some MS patients having extremely high mean PP_Resp_ value also had notably high mean PP_Card_ and mean PP_VLF_ values (data not shown).

**Figure 6.**
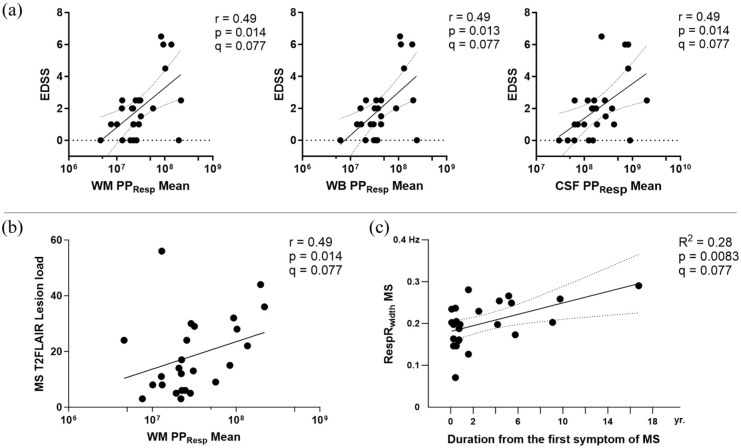
The correlation of respiratory pulsation power with EDSS and T2FLAIR lesion quantity: (a, b) Spearman correlations and best fitting curves (semi-logarithmic line, least squares method with 95% CI in (a), Poisson regression in (b)), of EDSS against mean PP_Resp_ in WM, WB and CSF (a) and MS T2FLAIR lesion load against mean PP_Resp_ in WM (b), and (c) the linear least squares regression curve, with 95% CI, of RespR_width_ and the disease duration of MS, that is, the duration since the first clinical symptom. Of note, the likely first symptom has been assessed retrospectively from the patient records by experienced neurologists after MS diagnosis has been set. The duration of 6 years or more from the first symptom in this sample reflects confirmed optic neuritis or nonspecific sensory symptoms, which have occurred years before MS diagnostic criteria, and clinically evident disease, were met. Note logarithmic *x*-axis in (a, b), *x*-values represent the mean MREG signal power in respiratory bandwidth. CI: confidence interval; MS: multiple sclerosis; FLAIR: fluid-attenuated inversion recovery; EDSS: Expanded Disability Status Scale; WB: whole brain; WM: white matter; CSF: cerebrospinal fluid; PP: power of pulsation; Resp: respiratory frequency band; RespR_width_: wider respiratory spectral component width, that is, respiratory rate variability range; MREG: magnetic resonance encephalography.

As depicted in Figure 6, mean PP_Resp_ in MS WB, WM and CSF (only the lateral ventricles included) significantly correlated with EDSS (WB *r* = 0.49 and *p* = 0.01, *q* = 0.077; WM *r* = 0.49 and *p* = 0.01, *q* = 0.077; CSF *r* = 0.49 and *p* = 0.01, *q* = 0.077) and MS T2FLAIR lesion quantity in WM (*r* = 0.49 and *p* = 0.01, *q* = 0.077). Similar correlations (*r*) of T2FLAIR lesion quantity and PP_Resp_ in WB and CSF were not statistically significant after correcting for multiple comparisons (FDR) as *q*-values were 0.16 and 0.17, respectively. In our early-stage MS sample, the MS T2FLAIR lesion quantity did not correlate with EDSS. There were no significant Spearman correlations between mean PP_Resp_ and HR, duration since the diagnosis, duration from the first symptom, age or mean absolute and mean relative head displacements.

### Increased respiratory spectral component width correlates with MS disease duration

In addition to brain areas with increased PP_Resp_, the variability range of respitory rate (RespR_width)_ determined from the scanner’s respiration belt data was increased in MS (mean = 0.20 Hz, SD = 0.052 Hz) compared to controls (mean = 0.16 Hz, SD = 0.059 Hz; *p* < 0.01; unpaired two-tailed *t*-test, *t* = 2.69, *q* = 0.033) while HR_width_ did not significantly differ (Figure 2(b)). RespR_width_ had a linear relationship with the duration of MS in linear regression analysis (*R*^2^ = 0.28 and *p* = 0.0083, *q* = 0.077; Figure 6(c)). RespR_width_ did not correlate with MS T2FLAIR lesion quantity, EDSS, age or RespR.

### Effects of covariates and MS clinical characteristics to the power of respiratory pulsation

RespR_width_ itself and smoking status did not have statistically significant independent effects on PP_Resp_ in the contrast between MS and control groups in a voxel-wise analysis. In our patient sample and in the independent voxel-wise statistical analysis, PP_Resp_ did not significantly differ in any brain areas between patients with and without additional neurological or psychiatric diseases, CNS targeting medication, brainstem lesion, relapse within 3 months prior to MREG scanning, or between patients having higher and lower EDSS (cutoff 2.0) or T2FLAIR lesion load (cutoff 15).

## Discussion

We non-invasively investigated physiological brain pulsations in MS over the whole brain, which revealed a novel pathophysiological finding. We detected significantly increased power of respiratory pulsations (PP_Resp_) widespread in the brain and, predominantly, in the CSF regions (Figures 2 and 4). This is direct evidence on dysfunction of CSF movement in MS. We detected neither arterial cardiovascular nor vasomotor VLF frequency pulsation power abnormalities and mean PP_Resp_ did not correlate with HR. Our correlation analyses indicate greater quantified neurological disability and radiological tissue damage with increasing PP_Resp_ (Figures 4 and 6). Of note, there was an increased autonomic variability range of respiratory rate, in positive correlation to MS duration (Figure 6(c)).

MREG signal pulsations at respiratory rate (~0.3 Hz) originate from CSF flow and intravenous blood outflow of deoxyhaemoglobin driven BOLD changes induced by respiratory pressure changes inside thorax and spinal canal.^26,33,39,40,43^ The respiratory pulsations are also present in the CSF spaces (that contain no (de)oxyhaemoglobin). There they are likely related to T1 and T2(*) phase effects of pulsatile water movement and time-of-flight effects, rather than any susceptibility weighted BOLD signal effects.^43,45^ Pulsatile flow effects have been demonstrated in phantom studies where MREG captures both water flow and the frequency of pulsatile water flow.^46,47^ Thus, the FFT biometrics of MREG signal do reflect flow-related pulsation power, originating from brain fluid dynamics. For more details on the origins of the MREG signal, see earlier publications.^34,43^

### Increased power of respiratory pulsation with respect to slower fMRI studies in MS

ALFF studies in MS have reported inconsistent results in different brain areas. Some studies report increased ALFF, while others report decreased ALFF.^61–67^ MS has been studied intensively with resting state functional connectivity (FC), through correlation of BOLD signal changes between different brain regions, giving conflicting results.^37,63,68–73^ In these ALFF studies, 0.08 or 0.1 Hz frequencies were analysed, and the repetition time in FC studies has been around 2000 ms. Both are significantly slower than physiological respiratory or cardiac rates or compared to the 10 Hz sampling rate afforded by MREG. In many earlier fMRI studies, it was not possible to assess simultaneously the entire brain. Sampling rate has a significant effect on fMRI metrics; according to Nyquist Theorem, sampling rate should be twice the frequency of a phenomenon under investigation.^74^

We show that most BOLD signal oscillation differences in MS compared to healthy controls occur at the respiratory frequency, which is usually in the range of 0.1–0.4 Hz. However, our data indicate that respiratory pulsations of some individuals may occur in part at a frequency <0.1 Hz, which could be detected with a slower sampling rate, thereby explaining inconsistency in results between previous ALFF and FC studies.

### Anatomy and clinical relevance of the respiratory pulsation power increase in MS

MS lesions tend to coincide with abnormal veins^3–6^ and respiration is, interestingly, a major driver of CSF movement and cerebral venous blood flow.^25,75,76^ In the MS group, we observed increased PP_Resp_ in grey matter, subcortical regions, periventricular white matter, medulla, cerebellum and, importantly, in the ventricles and other CSF spaces (Figures 2 and 4). As respiratory MREG signal oscillations are dominant in venous and CSF-filled spaces,^26^ the increased PP_Resp_ must originate in part from altered CSF dynamics in perivenous spaces of brain tissue. These results indicate marked increases in the driving forces of both venous blood flow and the perivascular, especially perivenous, CSF movement in conjunction with MS pathology.

Overall, heightened PP_Resp_ appears linked to CSF pulsation strength in early MS patients. Individual MS patients displayed substantial PP_Resp_ values >6 standard deviations (*Z*-score) above normal controls, with particularly high values in central and cortical CSF spaces (Figure 4). Notably, zones of increased PP_Resp_ did not overlap with T2FLAIR-hyperintense MS lesions to visual inspection, warranting more in-depth quantitative investigation. Interestingly, previous work links CSF pulse pressure and other intracranial pulsatility variables to brain volume loss and ventriculomegaly,^77^ and shows that the progression of MS leads to brain atrophy.^78^

Consistent with the previous findings, we demonstrated that increased relative CSF volume is associated with increased respiratory and cardiovascular brain pulsations. Thus, powered up pulsations of CSF may lead to atrophy or vice versa. Therefore, atrophy may contribute to the positive correlations between heightened respiratory pulsations and EDSS. Of note, atrophy can artefactually increase measured PP in regions near CSF due to partial volume effects. However, we showed that heightened PP_Resp_ in MS compared to the controls has also other mechanisms than co-located atrophy. The CSF volume did not differ between the groups. Thus, local CSF volume increase itself, such as larger ventricles or sulci due to atrophy, and their higher pulsation power on average, cannot explain the increased PP_Resp_ in the MS group compared to the controls. The total volume of potential WM atrophy was relatively low, and it had only 2.4% volumetric overlap with PP_Resp_ differences (Figure 2 and S1 Figure). That observed atrophy, that is, lower intensity T1 signal, in VBM analysis could alternatively be caused by darker native image T1 signal due to local MS lesions, since they are hypointense in T1 images, or true brain volume loss.

EDSS score, a measure of disability in MS, and respiratory pulsation power (PP_Resp_) showed a positive linear correlation in several brain regions, including the bilateral sensorimotor network and thalamus (Figure 5). Given that EDSS heavily weights walking ability, motor and sensory functions, and theoretically reflects brainstem-controlled autonomous nervous system functions, increased PP_Resp_ in MS presents a neuroanatomically relevant marker for abnormal brain function causing symptoms.

Notably, we observed increased PP_Resp_ in the MS group pontine (pneumotaxic) and medullary respiratory centers, which are precisely imaged in Raitamaa et al.,^79^ and intervening structures (Figure 2(b)). Importantly, and measured from *both* the respiration belt and MREG power spectra, MS patients in this sample exhibited abnormally broad autonomous variability range of respiratory rate, which may suggest the presence of autonomous nervous system dysfunction related to the longer disease duration (Figures 1 and 6). Heightened respiratory pulsations and disturbed brain perivascular/perivenous hydrodynamics may progressively perturb autonomous respiratory control over the course of the disease or vice versa.

We detected a zone of significant correlation between neurologic disability (EDSS) and PP_Resp_ lying *between* the pons pneumotaxic and apneustic centers (Figure 5), which further indicates alterations in respiratory physiology along with disease severity. Moreover, MS patients frequently show deterioration of respiratory function, and breathing-associated complications are among the most common causes of mortality in MS.^80^ Local demyelination or inflammation appears not to cause disturbed brainstem respiratory pulsation alone since we did not detect significant PP_Resp_ differences between MS patients with and without T2FLAIR hyperintense brainstem lesions.

Moreover, we saw that MS brain tissue damage co-join with pulsatile driving forces of the venous and CSF flows, insofar as MS T2FLAIR lesion quantity positively correlated with brain respiratory pulsation power (PP_Resp_) independently of EDSS. It is unlikely that extensive lesion burden of few subjects accounted for the observed respiratory pulsation differences between MS and control group as no voxel-wise PP_Resp_ difference were found between patients with higher and lower lesion load.

Overall, we found that MS is strongly related to local alterations in the pulsations driving CSF flow. Our present results also support earlier evidence of altered CSF circulation in MS, namely findings of increased CSF flow towards cranium that correlates moderately with lesion load and ventriculomegaly.^81^ Moreover, we note an interesting case report of an MS patient who benefitted from CSF shunting.^82^ The present results may further be related with findings of reduced CSF-mediated tissue clearance.^83^

### Pathophysiological origin of the respiratory pulsation power increase

Heightened respiratory pulsations likely have pathophysiological relevance in MS. Respiration significantly influences both CSF flow and venous blood flow.^75^ During breathing, the decreasing intrathoracic pressure drains internal jugular veins and the superior sagittal sinus,^76^ while a compensatory flow of CSF proceeds from the spinal cavity towards the cranium^75^ to maintain the constant intracranial volume of the brain, CSF and blood.^84^ Thus, the present finding of respiratory alterations in MS are coupled to both CSF dynamics and cerebral venous blood flow, and that is, also reflected in autonomous respiratory centers as increased PP_Resp_. Indeed, the pulsations were heightened in both CSF areas and brain tissue. Heightened power of respiratory pulsation is likely caused by several mechanisms. We propose that cardiovascular and pulmonary function, brain volume and tissue ‘stiffness’ generally affect the movement of blood, CSF, ISF and impulse propagation in the brain. Locally heightened brain pulsations suggest a presence of local abnormality of tissue and/or fluid dynamics, or compensatory increase in normally functioning areas.

Pioneering research indicates that macroscopic CSF-mediated brain solute clearance can be reduced in MS^83^ and that respiratory and vascular pulsations seem to have a role in CSF solute transportation^27,30,85,86^ in rats. Furthermore, the coupling of the vascular pulsation and ISF/CSF pulse into venous blood from the perivenous space has been observed.^87^ There is also evidence on perivenous fluid efflux system also in humans.^88,89^ Furthermore, the heightened respiratory brain pulsations in MS, could partially cause or arise from locally increased (or decreased) motion of fluid in the perivascular space. However, it remains still uncertain which specific (patho)physiological mechanisms are involved in local pulsation perturbation and to which extent each of them affects the power of pulsation.

In MS rat model, respiration (mechanical ventilation) was the main driver for leptomeningeal T-cells to transport in the CSF, rather than cardiac pulsations from the periarterial route, and immune cells have been detected to migrate around veins.^90,91^ Stalling of perivascular flow could conceivably cause local aggregations of immune cells, resulting in inflammatory cuffs or vice versa. Such perivascular cuffs are frequently evident in normal appearing white matter, demyelinating and demyelinated tissue in MS.^18–20^

Studies have demonstrated a loss of the normally polarised expression of AQP4 on astrocytic endfeet in the *glia limitans* of perivascular spaces in the vicinity of demyelinated MS lesions,^13^ and also in inflammatory cuffs in MS model mice,^92^ while AQP4 is over expressed in plaque and peri-plaque areas.^12,13^ Such alterations of AQP4 expression predisposes the tissue to localised oedema. An accumulation of ISF and associated solutes leads to increased T2 and FLAIR signals in MRI, which one should properly consider a direct sign of reduced solute/water transport *from* the oedemic tissue. Interestingly, MREG captures pulsatile waterflow^46,47^ but most lesions did not co-localise with increased power of pulsation, which is preliminarily suggested by *Z*-maps of six subjects, cf. Figure 4. Of note, AQP4 is under expressed in certain lesion stages.^11^

MS lesions typically form around veins in brain areas with poor perfusion.^7^ However, local blood perfusion tends to increase just before BBB breakdown and changes in tissue water diffusion, along with molecular magnetisation transfer changes.^10,93^ Upon loss of BBB integrity, there is an increase in tissue contrast enhancement centrifugal from the involved vein.^18,93^ Disease activity and damaged BBB, reflected by gadolinium enhancing lesions, likely affects perivenous fluid motion and PP_Resp_. However, our sample of MS patients with an enhancing lesion is too small for statistical analyses.

While heightened respiratory pulsations may serve to increase perivascular CSF movement or modulate venous blood flow, in case of blood vessel or BBB damage, failed/reduced solute convection via perivenous ducts or increased AQP4 expression, the increased power of respiratory pulsations may rather exacerbate the leakage of perivenous CSF and plasma, with potentially harmful molecules, *into* the tissue. For example, MS CSF samples show elevated levels of certain ceramides, which can evoke neuronal oxidative and mitochondrial damage.^94^ This kind of reversed fluid dynamics could theoretically contribute to oedematous T2 signal increase and later gliosis, along with demyelination and inflammation.

### Limitations and future directions

While our final sample of 25 non-medicated, good quality MS patient data is relatively small, it is generally considered adequate for fMRI analyses. Importantly, controls were carefully matched for sex and age to remove confounding effects. In statistical brain images with near-threshold *p* values, longer scanning or larger sample would better reveal any changes in vasomotor pulsation power. On the other hand, MREG dataset comprises 2961 brain volumes/subject, which is 20–30 times more than in conventional fMRI data, and offers five to six times more statistical power for inferences. In addition, fast sampling with verified bandpassing removes the aliasing of physiological noise over each other and enables precise inference of pulsation power.

Importantly, the effect of a few subjects with substantial disease burden or comorbidities is not large enough to cause our group level PP_Resp_ findings, which rather derive from our covariate analyses. Though, some covariates that we tested could have a significant effect on brain pulsations in a larger sample. We preferred larger sample size over strictly minimising all covariates in the patient population, in order to balance between type I and II error.

The present methods limit quantification of direction of the net fluid movement. The limited spatial resolution of present methods prevents the differentiation of increased PP_Resp_ between veins, perivascular spaces and cortical CSF spaces. In vivo microscopic studies might better elucidate the physiological effects of respiratory pulsations at a mechanistic level in future MS models.

## Conclusions

In summary, altered perivascular and intravascular fluid dynamics, along with damaged perivenous CSF conduits and structural brain and vascular tissue abnormalities, likely explain heightened respiratory brain pulsations in MS patients. Our findings preliminarily support the idea that intervention on perivenous fluid dynamics could affect the clinico-radiological appearance of the disease, contingent on confirmation of a causal relationship.

## Supplemental Material

sj-docx-1-jcb-10.1177_0271678X261453802 – Supplemental material for Heightened respiratory brain pulsations indicate fluid dynamic dysfunction in early multiple sclerosis Supplemental material, sj-docx-1-jcb-10.1177_0271678X261453802 for Heightened respiratory brain pulsations indicate fluid dynamic dysfunction in early multiple sclerosis by Jere Haverinen, Mervi Ryytty, Harri Rusanen, Johanna Krüger, Mika H Martikainen, Janne Kananen, Lauri Raitamaa, Niko Huotari, Matti Järvelä, Johanna Tuunanen, Heta Helakari, Emma Hiukka, Vesa Korhonen and Vesa Kiviniemi in Journal of Cerebral Blood Flow & Metabolism

sj-xlsx-2-jcb-10.1177_0271678X261453802 – Supplemental material for Heightened respiratory brain pulsations indicate fluid dynamic dysfunction in early multiple sclerosis Supplemental material, sj-xlsx-2-jcb-10.1177_0271678X261453802 for Heightened respiratory brain pulsations indicate fluid dynamic dysfunction in early multiple sclerosis by Jere Haverinen, Mervi Ryytty, Harri Rusanen, Johanna Krüger, Mika H Martikainen, Janne Kananen, Lauri Raitamaa, Niko Huotari, Matti Järvelä, Johanna Tuunanen, Heta Helakari, Emma Hiukka, Vesa Korhonen and Vesa Kiviniemi in Journal of Cerebral Blood Flow & Metabolism
