# Supplementary material for: Heightened respiratory brain pulsations indicate fluid dynamic dysfunction in early multiple sclerosis
Source: J Cereb Blood Flow Metab. 2026 May 28:0271678X261453802. Online ahead of print. doi: 10.1177/0271678X261453802 (PMC13328110; doi:10.1177/0271678X261453802)
Supplement: sj-docx-1-jcb-10.1177_0271678X261453802 – Supplemental material for Heightened respiratory brain pulsations indicate fluid dynamic dysfunction in early multiple sclerosis [file sj-docx-1-jcb-10.1177_0271678X261453802.docx]

**Heightened respiratory brain pulsations indicate fluid dynamic dysfunction in early multiple sclerosis**

Supporting Information

Jere Haverinen, Mervi Ryytty, Harri Rusanen, Johanna Krüger, Mika H. Martikainen, Janne Kananen, Lauri Raitamaa, Niko Huotari, Matti Järvelä, Johanna Tuunanen, Heta Helakari, Emma Hiukka, Vesa Korhonen, Vesa Kiviniemi

Corresponding author Vesa Kiviniemi, [vesa.kiviniemi@oulu.fi](mailto:vesa.kiviniemi@oulu.fi)

Contents

Methods

- *MREG sequence, data preprocessing and analysis*

Results

- *S1 Fig*

Brain areas with significantly increased power of respiratory pulsation in MS compared to healthy controls.

- *S2 Fig*

Brain areas with significant positive linear relationship between increased power of respiratory pulsation, cardiovascular (i.e. arterial frequency) pulsation and relative CSF volume increase in MS.

- *MREG and cardiorespiratory monitoring data correlation*
- *S3 Fig*

Correlations between scanner physiological measurements and MREG

References

Supplementary Source Data (a separate file)

Methods

*MREG sequence,* *data preprocessing and analysis*

MREG (Magnetic Resonance Encephalography) sequence was applied with a Siemens MAGNETOM Skyra 3T magnetic resonance imaging (MRI) scanner (Siemens Healthineers AG, Germany) equipped with a 32-channel head coil, except for one MS patient and the corresponding control who were scanned using a Siemens MAGNETOM Vida 3T MRI scanner with a 64-channel head coil.

MREG is a T2*-weighted 3D single shot stack of spirals (SOS) sequence that under-samples k-space to reach a sampling rate of 10 Hz and acquires the entire brain volume with single excitation, thus allowing functional imaging of physiological pulsations.^1,2^ The SOS gathers k-space in 60 ms bins with spiral in/out repeating in every other turn continuously in the positive z-direction, thus minimising the air-sinus off-resonance artifact.^1^ The point spread function of the SOS-sequence is 3 mm, with lesser off-resonance effects compared to other k-space undersampling strategies such as concentric shells and spokes.^1,3^ Scanning parameters for the 3D whole brain MREG sequence were repetition time (TR) 100 ms, echo time (TE) 36 ms, flip angle = 25°, 3D matrix = 64^3^, FOV = 192 mm with voxel size of 3 × 3 × 3 mm^3^. Furthermore, the magnetisation spoiling gradient between scans was set to 0.1 to avoid signal masking by stimulated echo drifts, while retaining sensitivity to physiological pulsations. For anatomical T1-weighted 3D MPRAGE, the parameters were TR = 1900 ms, TE = 2.49 ms, TI = 900 ms, flip angle = 9°, FOV = 240 mm, 0.9 mm cubic voxel.

MREG data were reconstructed using L2-Tikhonov regularisation with lambda 0.1, where the latter regularisation parameter was determined by the L-curve method with a MATLAB recon-tool from the sequence developers, resulting in an effective spatial resolution of 4.5 mm anisotropic.^1,4^ Conjugate gradient optimisation was performed using 35 iterations for increased robustness in the convergence of the images. Image reconstruction included a dynamic off-resonance in the k-space method (DORK), which importantly corrected for scanner warming and respiration-induced dynamic B_o_-field changes before preprocessing.^5,6^ Due to the high temporal resolution of MREG, physiological signal components may be precisely detected without aliasing.^7–10^ T1-relaxation effects were minimised by deleting the 8 s from the beginning of each scan.

The data were preprocessed with the standard FSL (Functional Magnetic Resonance Imaging of the Brain's software library) pipeline^11^, using high-pass filtration with a cut-off frequency of 0.008 Hz (125 s). FSL BET was used for brain extraction with neck clean-up and bias field correction.^12^ Motion correction was performed using FSL MCFLIRT software.^13^ Data were spatially smoothed with 5 mm full-width at half-maximum Gaussian kernel. The anatomical 3D MPRAGE images were used to register MREG data into Montreal Neurological Institute (MNI152) standard space using FSL FLIRT.^11,14^ Further calculations for power spectrum analysis of physiological MREG signals were performed using FSL and AFNI (Analysis of Functional NeuroImages).^15^ Corresponding scanner respiratory belt and finger SpO_2_ photoplethysmograph data spectra were calculated in MATLAB (The MathWorks Inc, MA, USA) using a fast Fourier transform algorithm. All calculated images were aligned and, to exclude non-brain voxels, then masked with an isotropic 3 mm MNI152 brain template.

Since this study focuses on the sources of physiological BOLD (blood oxygenation level-dependent) signals, we intentionally retained as much as possible the physiological pulsations in the data. Therefore, CSF, white matter and global signals were not regressed from the datasets.

Results


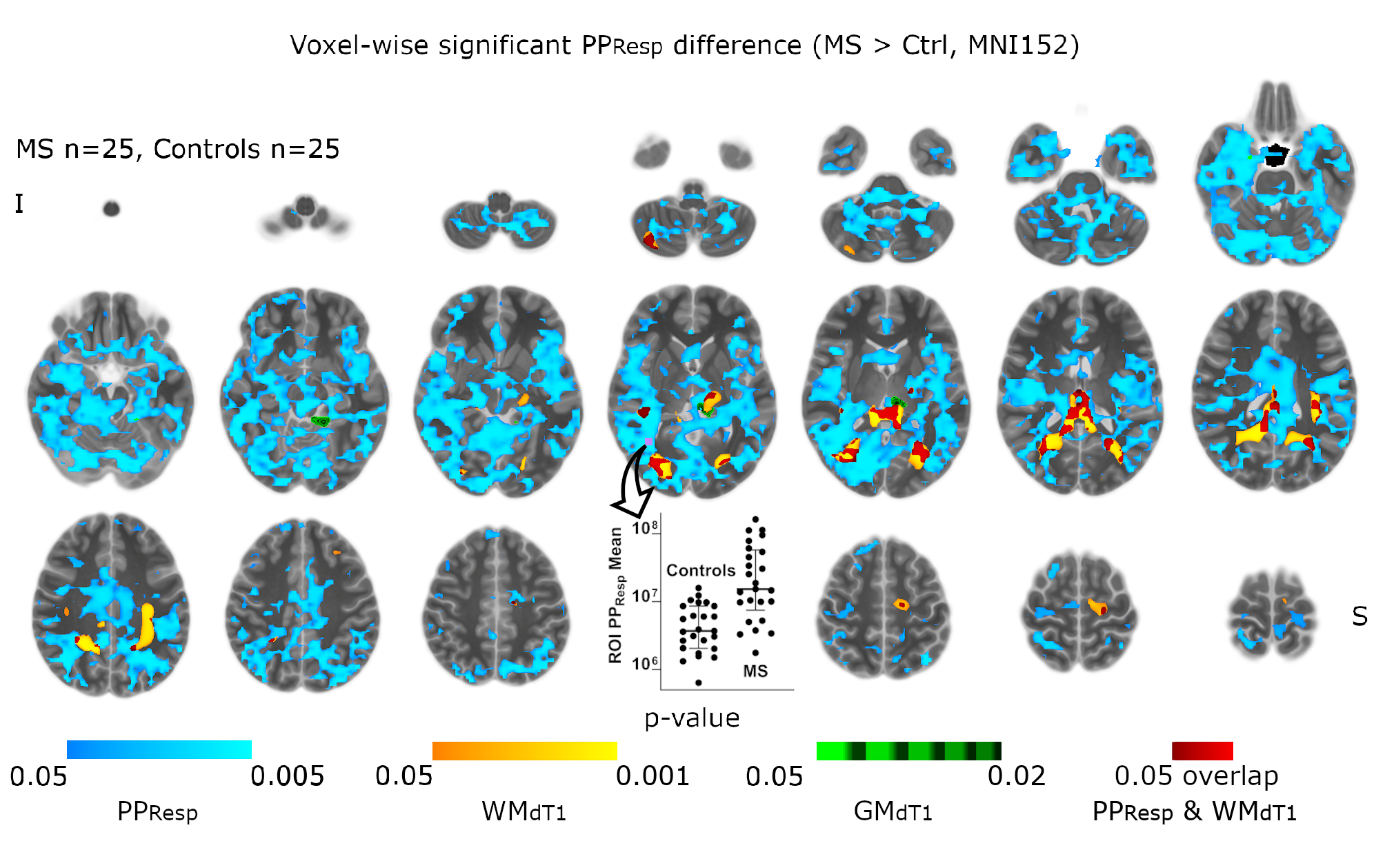


**S1 Fig. Brain areas with significantly increased power of respiratory pulsation in MS compared to healthy controls.** Axial image slices show voxels with significantly increased power of respiratory frequency pulsation in MS, with adjustment for mean relative head displacement. This is the same statistical image as the main Figure 2 B, but showing more horizontal slices. Background: MNI152 standard template. MS = multiple sclerosis, Ctrl = control, PP = power of pulsation, Resp = respiratory frequency band, WM_dT1_ = white matter atrophy or T1 signal hypointensity in MS > controls, GM_dT1_ = grey matter atrophy or T1 signal hypointensity in MS > controls, I = inferior, S = superior.


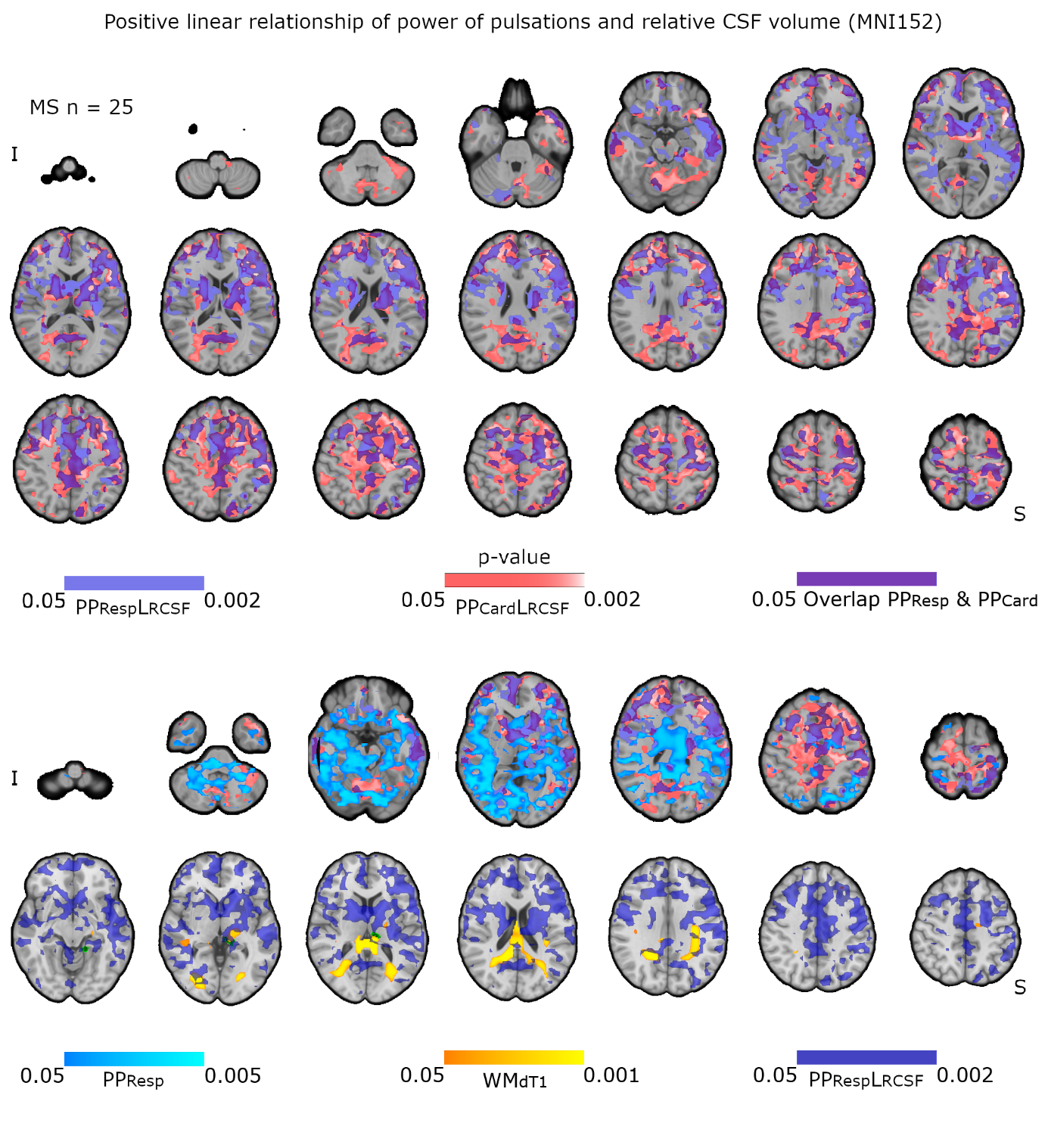


**S2 Fig.** **Brain areas with significant positive linear relationship between increased power of respiratory pulsation, cardiovascular (i.e. arterial frequency) pulsation and relative CSF volume increase in MS.** These are the same statistical images as the main Figure 2 B and 3, but showing more horizontal slices. Increased CSF volume in the ventricles and sulci compared to brain tissue volume, i.e. increased relative CSF volume, has positive linear relationship with heightened respiratory and cardiovascular arterial pulsations in MS, and often in same brain regions. This relationship is evident widely in the brain but there is only moderate overlap with areas where PP_Resp_ is significantly increased compared to healthy controls. The linear positive relationship between relative CSF volume and PP_Resp_ is only minimally evident in atrophied brain areas, i.e. in areas where the MS patients in our sample had more hypointense T1 signal compared to the healthy controls. Background: MNI152 standard template. MS = multiple sclerosis, PP = power of pulsation, Resp = respiratory frequency band, Card = cardiovascular (i.e. arterial) frequency band, L_RCSF_ = positive linear relationship with relative CSF volume. WM_dT1_ = white matter atrophy or T1 signal hypointensity in MS > controls, CSF = cerebro spinal fluid, I = inferior, S = superior.

*MREG and cardiorespiratory monitoring data correlation*

Correlations of cardiorespiratory frequencies from the monitoring data (scanner respiration belt and finger SpO_2_ photoplethysmograph (PPG)) FFT (fast Fourier transform) spectra against respective MREG data were calculated with Spearman correlation coefficients. MREG cardiorespiratory frequencies correlated precisely with the monitoring data, i.e., r = 0.88 – 0.99, *p* = 0.0006 (see S2 Fig). P-values were family-wise error corrected with Holm-Šídák method, *alpha* = 0.01. To get true respiratory and heart rate values for data analysis, we carefully avoided heterodyne peaks (cardiorespiratory envelope modulation) and harmonic peak frequencies. ^10^ The presence of heterodynes and harmonic frequencies are evident in the main Figure 1 G.


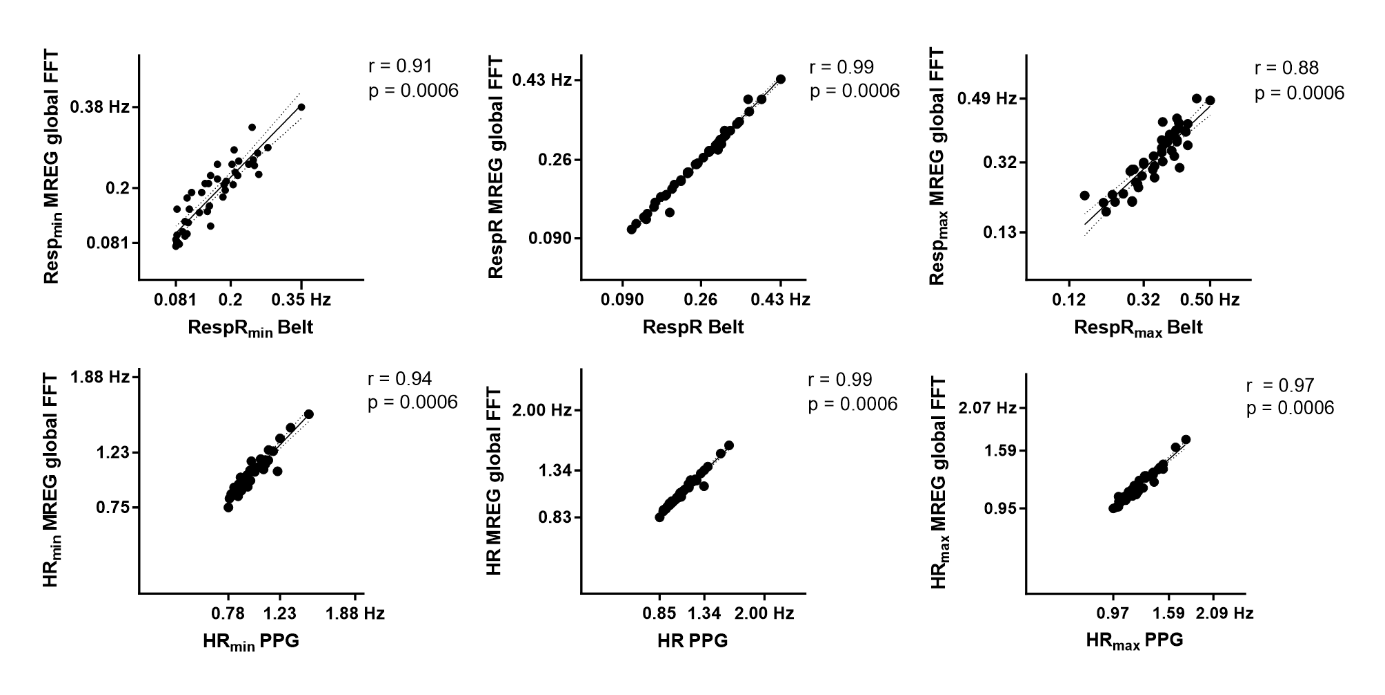
**S3 Fig. Correlations between scanner physiological measurements and MREG.** Spearman correlations of cardiorespiratory monitoring data and MREG, along with the linear regression curve with 95% CI. Cardiorespiratory monitoring data of three MS patients and five controls was corrupted, such that their RespR and HR were not verified from external measurements. CI = confidence interval, MREG = Magnetic Resonance Encephalography RespR = principal respiratory rate, HR = principal heart rate, ---_min_ = minimum frequency power above baseline noise, ---_max_ = maximum frequency power above baseline noise, PPG = photoplethysmograph

References

1. Assländer J, Zahneisen B, Hugger T, et al. Single shot whole brain imaging using spherical stack of spirals trajectories. *Neuroimage* 2013; 73: 59–70.

2. Hennig J, Kiviniemi V, Riemenschneider B, et al. 15 Years MR-encephalography. *Magnetic Resonance Materials in Physics, Biology and Medicine* 2021; 34: 85–108.

3. Zahneisen B, Hugger T, Lee KJ, et al. Single shot concentric shells trajectories for ultra fast fMRI. *Magn Reson Med* 2012; 68: 484–494.

4. Hugger T, Zahneisen B, LeVan P, et al. Fast undersampled functional magnetic resonance imaging using nonlinear regularized parallel image reconstruction. *PLoS One*; 6. Epub ahead of print 14 December 2011. DOI: 10.1371/JOURNAL.PONE.0028822.

5. Pfeuffer J, Van de Moortele P-F, Ugurbil K, et al. Correction of physiologically induced global off-resonance effects in dynamic echo-planar and spiral functional imaging. *Magn Reson Med* 2002; 47: 344–353.

6. Zahneisen B, Assländer J, LeVan P, et al. Quantification and correction of respiration induced dynamic field map changes in fMRI using 3D single shot techniques. *Magn Reson Med* 2014; 71: 1093–1102.

7. Kiviniemi V, Wang X, Korhonen V, et al. Ultra-fast magnetic resonance encephalography of physiological brain activity – Glymphatic pulsation mechanisms? *Journal of Cerebral Blood Flow & Metabolism* 2016; 36: 1033–1045.

8. Rajna Z, Mattila H, Huotari N, et al. Cardiovascular brain impulses in Alzheimer’s disease. *Brain* 2021; 144: 2214–2226.

9. Huotari N, Raitamaa L, Helakari H, et al. Sampling Rate Effects on Resting State fMRI Metrics. *Front Neurosci*; 13. Epub ahead of print 2 April 2019. DOI: 10.3389/fnins.2019.00279.

10. Raitamaa L, Huotari N, Korhonen V, et al. Spectral analysis of physiological brain pulsations affecting the BOLD signal. *Hum Brain Mapp* 2021; 42: 4298–4313.

11. Jenkinson M, Beckmann CF, Behrens TEJ, et al. FSL. *Neuroimage* 2012; 62: 782–790.

12. Smith SM. Fast robust automated brain extraction. *Hum Brain Mapp* 2002; 17: 143–155.

13. Jenkinson M, Bannister P, Brady M, et al. Improved Optimization for the Robust and Accurate Linear Registration and Motion Correction of Brain Images. *Neuroimage* 2002; 17: 825–841.

14. Grabner G, Janke AL, Budge MM, et al. Symmetric Atlasing and Model Based Segmentation: An Application to the Hippocampus in Older Adults. 2006, pp. 58–66.

15. Cox RW. AFNI: Software for Analysis and Visualization of Functional Magnetic Resonance Neuroimages. *Computers and Biomedical Research* 1996; 29: 162–173.
